# PIK3CA mutations predict recurrence in localized microsatellite stable colon cancer

**DOI:** 10.1002/cam4.370

**Published:** 2015-02-02

**Authors:** Gilles Manceau, Laetitia Marisa, Valérie Boige, Alex Duval, Marie-Pierre Gaub, Gérard Milano, Janick Selves, Sylviane Olschwang, Valérie Jooste, Michè le Legrain, Delphine Lecorre, Dominique Guenot, Marie-Christine Etienne-Grimaldi, Sylvain Kirzin, Laurent Martin, Come Lepage, Anne-Marie Bouvier, Pierre Laurent-Puig

**Affiliations:** 1 Unité Mixte de Recherche S1147, Paris Sorbonne Cité, Université Paris Descartes, INSERMParis, France; 2 Assistance Publique-Hôpitaux de Paris, Service de Chirurgie Digestive et Hépato-Bilio-Pancréatique, Hôpital Pitié-SalpêtrièreParis, France; 3 Institut Universitaire de Cancérologie, Université Pierre et Marie Curie-Paris 6Paris, France; 4 “Cartes d'Identité des Tumeurs” Program, Ligue Nationale Contre le CancerParis, France; 5 Institut Gustave RoussyVillejuif, France; 6 Unité Mixte de Recherche S938, Centre de Recherche Hôpital Saint-Antoine, INSERMParis, France; 7 Laboratoire de Biochimie et Biologie Moléculaire, Hôpitaux Universitaires de Strasbourg, Hôpital de HautepierreStrasbourg, France; 8 EA 3430 Progression tumorale et microenvironnement. Approches translationnelles et Epidémiologie. Fédération de Médecine Translationnelle de Strasbourg, Université de StrasbourgStrasbourg, France; 9 Laboratoire d'Oncopharmacologie EA 3836, Centre Antoine LacassagneNice, France; 10 Unité Mixte de Recherche 1037, Centre de Recherche en Cancérologie de Toulouse, Université de Toulouse III, INSERMToulouse, France; 11 Unité Mixte de Recherche S910, Faculté de Médecine La Timone, INSERMMarseille, France; 12 Pôle DACCORD, Hôpital La TimoneMarseille, France; 13 Département d'Oncologie, Hôpital ClairvalMarseille, France; 14 Département de Gastroentérologie, Hôpital Ambroise ParéMarseille, France; 15 Registre Bourguignon des Cancers Digestifs, INSERM U866CHU Dijon, France; 16 Service d'anatomie et de cytologie pathologiquesCHU Dijon, France

**Keywords:** Biomarker, colon cancer, microsatellite instability, mismatch repair, mutations, PIK3CA, prognosis

## Abstract

*PIK3CA*, which encodes the p110*α* catalytic subunit of PI3K*α*, is one of the most frequently altered oncogenes in colon cancer (CC), but its prognostic value is still a matter of debate. Few reports have addressed the association between *PIK3CA* mutations and survival and their results are controversial. In the present study, we aimed to clarify the prognostic impact of *PIK3CA* mutations in stage I–III CC according to mismatch repair status. Fresh frozen tissue samples from two independent cohorts with a total of 826 patients who underwent curative surgical resection of CC were analyzed for microsatellite instability and screened for activating point mutations in exon 9 and 20 of *PIK3CA* by direct sequencing. Overall, 693 tumors (84%) exhibited microsatellite stability (MSS) and 113 samples (14%) harbored *PIK3CA* mutation. In the retrospective training cohort (*n* = 433), patients with *PIK3CA*-mutated MSS tumors (*n* = 47) experienced a significant increased 5-year relapse-free interval compared with *PIK3CA* wild-type MSS tumors (*n* = 319) in univariate analysis (94% vs. 68%, Log-rank *P *= 0. 0003) and in multivariate analysis (HR = 0.12; 95% confidence interval, 0.029–0.48; *P *= 0.0027). In the prospective validation cohort (*n* = 393), the favorable prognostic impact of *PIK3CA* mutations in MSS tumors (*n* = 327) was confirmed (83% vs. 67%, Log-rank *P *= 0.04). Our study showed that *PIK3CA* mutations are associated with a good prognosis in patients with MSS stage I–III CC.

## Introduction

The phosphoinositide 3-kinase (PI3K)/AKT/mTOR signaling pathway is critical for cell growth, survival, and malignant transformation. It is inappropriately activated in many different cancer types 1. Activation is often mediated by mutations occurring in *PIK3CA*, which encodes the p110*α* catalytic subunit of a heterodimeric class IA PI3K called PI3K*α*. This gene is one of the most frequently mutated genes (16%) in colorectal cancer (CRC), after notably *TP53* (51%), *APC* (37%), and *KRAS* (36%) 2.

In the signal transduction, after ligand binding to a tyrosine kinase receptor, activated PI3K*α* phosphorylates phosphatidylinositol 4,5-bisphosphate (PIP2) at the 3′-position of the inositol ring, allowing the generation of phosphatidylinositol 3,4,5-triphosphate (PIP3). This second messenger binds and recruits the 3-phosphoinositide-dependent protein kinase-1 (PDK1), which thereafter activates AKT/PKB, a serine/threonine kinase involved in regulating many biological processes such as cell survival, growth, and metabolism 3. There are three major mutational hotspots (at codons 542, 545, and 1047) in exons 9 and 20 of *PIK3CA*, partially encoding the helical domain and the C-terminal kinase domain of the protein, respectively 4. Activating point mutations in these amino-acid residues elevate the enzymatic activity of PI3K and contribute to tumorigenesis through cell proliferation, decreased apoptosis and autophagy, loss of contact inhibition, induction of angiogenesis, and increased tumor invasion 5–7.

As one of the most commonly deregulated pathways in solid human cancers, targeting the PI3K/AKT/mTOR pathway could be of important therapeutic interest 8. Indeed, a number of PI3K or dual PI3K-mTOR inhibitors have been, or will soon be, introduced into clinical trials as antitumor agents for the treatment of CRC and other malignancies 9–11. Preclinical studies demonstrated that *PIK3CA* mutations predict response to these agents, presumably due to oncogene addiction 12–15. However, prognosis of CRC patients harboring *PIK3CA* mutation remains unclear and results from previous studies dealing with this issue seem conflicted 16–22. Thus, the prognostic role of *PIK3CA* as an independent predictor of recurrence and/or survival in patients with CRC remains to be determined.

The discrepancy of published results could be in part explained by the well-known molecular heterogeneity of CRC. Among the different individualized molecular subgroups, tumors with microsatellite instability (MSI) accounts for ∽15% of all CRCs and are characterized by defective DNA mismatch repair and genomic instability. The remaining 85% of microsatellite stable (MSS) CRC display chromosomal instability resulting in hyperploidy associated with allelic losses. Both groups show specific particularities in terms of natural history, tumor location, pathological features, mechanisms of carcinogenesis, and genetic mutation patterns 23.

The aim of this multicenter study was to investigate the prognostic value of *PIK3CA* mutations in nonmetastatic colon cancer (CC) according to MSI status.

## Material and Methods

### Study population

The French national “Cartes d'Identité des Tumeurs” (CIT) program involved a multicenter cohort of 782 patients with stage I–IV CRC who underwent surgery between 1987 and 2007 in seven centers. Fresh frozen primary tumor tissue samples were retrospectively collected. Clinicopathological data were extracted from the medical charts and centrally reviewed for all patients. This retrospective cohort was used as a training cohort.

For validation purpose, the prospective cohort from the population-based registry of digestive cancer in the Côte-d'Or area (Burgundy, France) previously describe elsewhere was used 24,25. Tumor samples from this validation patient cohort included all CC resected between 1998 and 2002 for which frozen tissue material was available and suitable for molecular analysis.

Patients with rectal cancer (located within 15 cm from the anal verge), distant metastasis or who received neoadjuvant therapy (either chemotherapy and/or radiotherapy) were excluded from analysis. Thus, only stage I–III CC according to the 7th edition of the AJCC/UICC tumor-node-metastasis (TNM) classification operated in a curative intent were further considered for evaluation 26. *PIK3CA* status had also to be determined for each sample. This study was approved by the Institutional Review Boards of all participating centers.

### Mutation analysis

Genomic DNA was extracted from fresh frozen tissues. Exons 9 and 20 of the *PIK3CA* gene were selected for screening by direct Sanger sequencing on both strands because of the high frequency of somatic mutations known to be clustered in these regions 4. All samples found to be mutated were PCR-amplified and sequenced in a second, independent experiment. PCR conditions for amplification and primer sequences are available upon request.

The seven most common somatic mutations of *KRAS* located within codon 12 (G12D, G12V, G12C, G12A, G12S, and G12R) and codon 13 (G13D), as well as *BRAF* V600E mutation, were assessed by allelic discrimination using TaqMan-specific probes as previously described 27.

### Microsatellite status and CpG island methylator phenotype analysis

In the retrospective CIT cohort, MSI status was assessed according to the panel of five microsatellites approved by the consensus conference (D2S123, D5S346, D17S250, BAT25, and BAT26) 28. In the validation cohort, MSI status was determined as previously described 29.

We used the MSP (gel-based methylation-specific PCR) method with the panel of the five markers CACNA1G, IGF2, NEUROG1, RUNX3, and SOCS1 to determine CpG island methylator phenotype (CIMP) status 30. After DNA bisulfite treatment, two multiplex methylation-specific PCR were performed. Capillary electrophoresis on automatic sequencer (ABI 3130 Genetic analyzer; Applied Biosystems, Foster City, CA, USA) was used for fragment analysis. The methylation status of each gene was determined as detailed by Weisenberg and colleagues 29. Methylator phenotype-positive cases (CIMP+) were defined as those with ≥3 methylated promoters and CIMP—cases as those with <2 methylated promoters.

### Statistic analysis

The distribution of patient and tumor characteristics was compared across cohorts by the Chi-squared test or the Fisher's exact test for categorical data, as appropriate, and by the Welch's *t*-test for continuous data.

Relapse-free interval (RFI) was defined as the time from CC resection to locoregional and/or distant recurrence, whichever came first. Patients alive with no evidence of disease at last follow-up and patients who died without any recurrence were censored. Overall survival (OS) was defined as the period of time between CC surgery and death. Survival curves were plotted according to the method of Kaplan and Meier and differences between survival distributions were assessed by the log-rank test. Univariate and multivariate models for survival analysis were computed using Cox proportional hazards regression. All the variables that were significant in univariate analysis were included in the multivariate model. The proportional hazards assumptions were tested to examine the appropriateness of the models.

All *P*-values were two-sided and statistical significance was assumed for *P* ≤ 0.05. All statistical analyses were performed using the *R* statistical environment (http://www.R-project.org). Survival analyses were performed using the *R* package survival.

## Results

### Patient characteristics

A total of 826 samples from stage I–III CC with successful mutation analysis for *PIK3CA* were obtained from both cohorts (*n* = 433 in the training cohort and *n* = 393 in the validation cohort). Patients’ characteristics are depicted in Table1. Overall, 133 tumors exhibited MSI phenotype (67 [15%] in the training cohort and 66 [17%] in the validation cohort, *P *= 0.67). Among MSS tumors, patients included in the validation cohort were significantly older (69 vs. 73 years, *P *= 0.00021), had less advanced stage (45% vs. 35% for stage III, *P *= 0.015) and were less likely to receive adjuvant chemotherapy (45% vs. 32%, *P *= 0.00071), as compared with the training cohort. Among MSI tumors, patients in the validation cohort were significantly older (74 vs. 80 years, *P *= 0.0016). Significant more patients had a Lynch syndrome in the training cohort (4% vs. 1%, *P *= 0.0087). Only one patient, included in the validation cohort, had a familial adenomatous polyposis.

**Table 1:** Clinical and pathological characteristics of patients according to microsatellite status in the two cohorts

Clinical or pathological features	All cases	*n* (%)	Total CIT (%)	Total Dijon (%)	*P* value	MSS CIT (%)	MSS Dijon (%)	*P* value	MSI CIT (%)	MSI Dijon (%)	*P* value
Gender											
Female	826	361 (44)	189 (44)	172 (44)	0.97	156 (43)	130 (40)	0.49	33 (49)	42 (64)	0.13
Male		465 (56)	244 (56)	221 (56)		210 (57)	197 (60)		34 (51)	24 (36)	
Age (years)^1^	826	826	69 [24–96]	73 [33–95]	1.6 × 10−6	69 [25–96]	73 [36–95]	0.00021	74 [24–92]	80 [33–91]	0.0016
Tumor location^2^											
Distal	826	472 (57)	249 (58)	223 (57)	0.88	234 (64)	214 (65)	0.74	15 (22)	9 (14)	0.28
Proximal		354 (43)	184 (42)	170 (43)		132 (36)	113 (35)		52 (78)	57 (86)	
TNM stage											
I	826	76 (9)	33 (8)	43 (11)	0.0052	25 (7)	35 (11)	0.015	8 (12)	8 (12)	0.23
II		431 (52)	211 (49)	220 (56)		175 (48)	176 (54)		36 (54)	44 (67)	
III		319 (39)	189 (44)	130 (33)		166 (45)	116 (35)		23 (34)	14 (21)	
Adjuvant CT											
No	823	540 (66)	257 (59)	283 (72)	0.00014	203 (55)	222 (68)	0.00071	54 (82)	61 (92)	0.12
Yes		283 (34)	175 (41)	108 (28)		163 (45)	103 (32)		12 (18)	5 (8)	
Associated syndrome											
FAP	806	1 (0)	0 (0)	1 (0)	0.0087	0 (0)	1 (0)	0.96	0 (0)	0 (0)	0.0002
Lynch syndrome		22 (3)	18 (4)	4 (1)		0 (0)	0 (0)		18 (35)	4 (6)	
None		783 (97)	395 (96)	388 (99)		362 (100)	326 (100)		33 (65)	62 (94)	

MSS, microsatellite stable; MSI, microsatellite instable; CT, chemotherapy; FAP, familial adenomatous polyposis.

1 Median [range].

2 Proximal colon included cecum, ascending colon, hepatic flexure, and transverse colon, and distal colon included splenic flexure, descending, and sigmoid colon.

### *PIK3CA* mutations analysis and associations with other molecular and clinicopathological features

Among the 826 tumors suitable for *PIK3CA*, 113 (14%) tumors displayed a mutation in exon 9 and/or 20 (59 in the training cohort and 54 in the validation cohort). Most *PIK3CA* mutations were located in exon 9 with 38 tumors in the training cohort (64% of the mutated samples) and 32 tumors in the validation cohort (59% of the mutated samples). *PIK3CA* mutations in exon 20 were detected in 6% of the tumors in both cohorts. Only four tumors (three MSS tumors and one MSI tumor, all in the training cohort) harbored *PIK3CA* mutation in both exons 9 and 20, which accounted for 0.5% of all studied samples and 4% of all mutated samples (Table2).

**Table 2:** Location of *PIK3CA* mutations according to microsatellite status in the two cohorts

*PIK3CA* mutant types	CIT cohort		Dijon cohort		CIT cohort vs. Dijon cohort	
	MSS tumors (%)	MSI tumors (%)	MSS tumors (%)	MSI tumors (%)	*P* value MSS tumors	*P* value MSI tumors
Exon 9 mutant	30 (64)	4 (33)	30 (70)	2 (18)	0.24	0.39
Exon 20 mutant	14 (30)	7 (58)	13 (30)	9 (82)		
Exon 9 and 20 mutant	3 (6)	1 (8)	0 (0)	0 (0)		

MSS, microsatellite stable; MSI, microsatellite instable.

The determination of *KRAS* and *BRAF* mutational status could be ascertained for 817 (99%) and 780 (94%) tumors, respectively (Table3). In the whole population, we identified *KRAS* mutation at codon 12 or 13 and V600E *BRAF* mutation in 37% and 11% of cases, respectively, with no statistical difference between the two cohorts, either globally or according to MSI status. As expected, *KRAS* and *BRAF* mutations were mutually exclusive. Concomitant *PIK3CA* and *KRAS* mutations were found in 55 tumors (7%), whereas only 11 tumors (1%) were mutated for both *PIK3CA* and *BRAF*.

**Table 3:** Molecular characteristics of colon cancers according to microsatellite status in the two cohorts

Molecular features	All cases	*n* (%)	Total CIT (%)	Total Dijon (%)	*P* value	MSS CIT (%)	MSS Dijon (%)	*P* value	MSI CIT (%)	MSI Dijon (%)	*P* value
*PIK3CA* status											
Mutant	826	113 (14)	59 (14)	54 (14)	0.96	47 (13)	43 (13)	0.99	12 (18)	11 (17)	0.97
Wild-type		713 (86)	374 (86)	339 (86)		319 (87)	284 (87)		55 (82)	55 (83)	
*KRAS* status											
Mutant	817	301 (37)	164 (38)	137 (35)	0.37	147 (40)	129 (40)	0.92	17 (27)	8 (12)	0.062
Wild-type		516 (63)	263 (62)	253 (65)		216 (60)	195 (60)		47 (73)	58 (88)	
*BRAF* status											
Mutant	780	85 (11)	35 (9)	50 (13)	0.12	7 (2)	12 (4)	0.37	28 (43)	38 (58)	0.11
Wild-type		695 (89)	353 (91)	342 (87)		316 (98)	315 (96)		37 (57)	27 (42)	
CIMP status											
CIMP−	763	606 (79)	301 (81)	305 (78)	0.37	278 (90)	288 (88)	0.68	23 (37)	17 (26)	0.26
CIMP+		157 (21)	71 (19)	86 (22)		32 (10)	38 (12)		39 (63)	48 (74)	

MSS, microsatellite stable; MSI, microsatellite instable; CIMP, CpG island methylator phenotype.

We assessed the relationship between *PIK3CA* mutation and clinicopathological and molecular features in each cohort according to MSI status (Table4). The frequency of *PIK3CA* mutations was not different between MSS and MSI tumors (13% vs. 18%, *P *= 0.36 in the training cohort and 13% vs. 17%, *P *= 0.57 in the validation cohort). Among MSS tumors, *PI3KCA* mutations were significantly more frequently located in the proximal colon and associated with CIMP-positive tumors in the training cohort (51% vs. 34%, *P *= 0.033 and 23% vs. 8%, *P *= 0.0063 respectively), and significantly more frequent in female in the validation cohort (56% vs. 37%, *P *= 0.032). A significant association between *PIK3CA* and *KRAS* mutations (58% vs. 37%, *P *= 0.014) was found in the validation cohort. Among MSI tumors, *PIK3CA* mutations were significantly more frequent in patients with early-stage disease in the training cohort (33% vs. 7% for stage I, *P *= 0.034).

**Table 4:** Clinical, pathological, and molecular characteristics of tumors according to *PIK3CA* and microsatellite status in the two cohorts

Features	MSS tumors						MSI tumors					
	CIT cohort		*P* value	Dijon cohort		*P* value	CIT cohort		*P* value	Dijon cohort		*P* value
	PIK3CAm	PIK3CAwt		PIK3CAm	PIK3CAwt		PIK3CAm	PIK3CAwt		PIK3CAm	PIK3CAwt	
Gender												
Female	18 (38)	138 (43)	0.63	24 (56)	106 (37)	0.032	6 (50)	27 (49)	0.79	7 (64)	35 (64)	0.73
Male	29 (62)	181 (57)		19 (44)	178 (63)		6 (50)	28 (51)		4 (36)	20 (36)	
Age (years)^1^	69 [25–96]	69 [37–96]	0.62	73 [49–94]	73 [36–95]	0.75	70 [32–88]	75 [24–92]	0.093	77 [67–88]	80 [33–91]	0.78
TNM.stage												
I	3 (7)	22 (7)	0.54	5 (12)	30 (11)	0.19	4 (33)	4 (7)	0.034	1 (9)	7 (13)	0.89
II	26 (55)	149 (47)		28 (65)	148 (52)		4 (33)	32 (58)		8 (73)	36 (65)	
III	18 (38)	148 (46)		10 (23)	106 (37)		4 (33)	19 (35)		2 (18)	12 (22)	
Tumor.location												
Distal	23 (49)	211 (66)	0.033	23 (53)	191 (67)	0.11	2 (17)	13 (24)	0.89	0 (0)	9 (16)	0.34
Proximal	24 (51)	108 (34)		20 (47)	93 (33)		10 (83)	42 (76)		11 (100)	46 (84)	
Adjuvant CT												
No	28 (60)	175 (55)	0.65	32 (74)	190 (67)	0.45	10 (83)	44 (81)	0.79	10 (91)	51 (93)	0.68
Yes	19 (40)	144 (45)		11 (26)	92 (33)		2 (17)	10 (19)		1 (9)	4 (7)	
*KRAS* status												
Mutant	22 (48)	125 (39)	0.36	25 (58)	104 (37)	0.014	6 (55)	11 (21)	0.053	2 (18)	6 (11)	0.87
Wild-type	24 (52)	192 (61)		18 (42)	177 (63)		4 (45)	42 (79)		9 (82)	49 (89)	
*BRAF* status												
Mutant	1 (2)	6 (2)	0.6	1 (2)	11 (4)	0.95	3 (27)	25 (46)	0.41	6 (55)	32 (59)	0.96
Wild-type	46 (98)	270 (98)		42 (98)	273 (96)		8 (73)	29 (54)		5 (45)	22 (41)	
CIMP status												
CIMP−	33 (77)	245 (92)	0.0063	37 (86)	251 (89)	0.8	5 (50)	18 (35)	0.57	3 (27)	14 (26)	1
CIMP+	10 (23)	22 (8)		6 (14)	32 (11)		5 (50)	34 (65)		8 (73)	40 (74)	

CT, chemotherapy; m, mutated; wt, wild-type. Figures in brackets represent the percentages.

1 Median [range].

### *PI3KCA* mutation and patient survival according to microsatellite status

We further examined the prognostic impact of *PIK3CA* mutations in nonmetastatic CC after curative resection. The median follow-up was 51 months (range, 1–192 months) in the training cohort and 61 months (range, 1–143 months) in the validation cohort. The 5-year RFI was similar in the training cohort and in the validation cohort, either globally (63% vs. 68%, *P *= 0.24), and also within MSS and MSI tumors (63% vs. 66%, *P *= 0.58 and 67% vs. 81%, *P *= 0.23, respectively).

**Figure 1:**
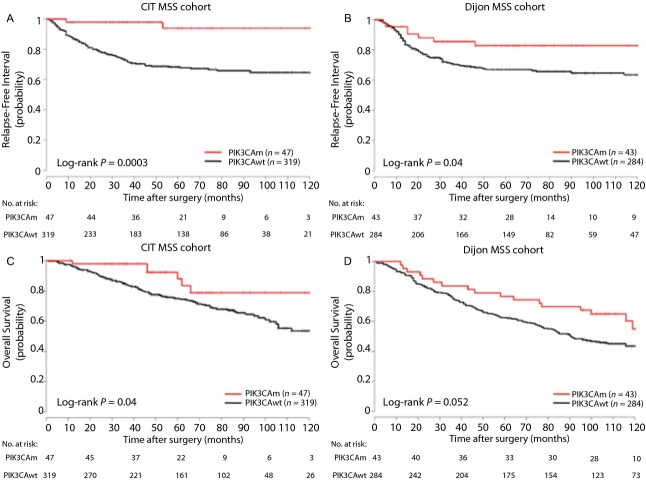
Kaplan–Meier curves for relapse-free interval in the CIT cohort (A) and in the Dijon cohort (B), for overall survival in the CIT cohort (C) and in the Dijon cohort (D) according to *PIK3CA* status in microsatellite stable tumors.

Within the MSS CC subgroup of the training cohort, patients with *PIK3CA-*mutated CC experienced a significantly higher RFI than those without *PIK3CA* mutation (5-year RFI 94% vs. 68%, Log-rank *P *= 0.0003; Hazard Ratio [HR] = 0.12; 95% confidence interval [CI], 0.029–0.48) on univariate analysis (Fig.1A). This finding was confirmed in the validation cohort (5-year RFI 83% vs. 67%, Log-rank *P* = 0.04; HR = 0.45; 95% CI, 0.21–0.97; *P *= 0.04) (Fig.1B). Similarly, OS was significantly higher in patients with MSS *PIK3CA-*mutated CC than those without *PIK3CA* mutation in the training cohort (5-year OS 88% vs. 75%, Log-rank *P *= 0.04; HR = 0.43; 95% CI, 0.19–0.98) (Fig.1C), and a strong tendency was also observed in the validation cohort (5-year OS 77% vs. 62%, Log-rank *P *= 0.052; HR = 0.61, 95% CI, 0.37–1) (Fig.1D). A subgroup analysis according to TNM stage (stage I–II and stage III) was also performed for RFI and OS in both cohorts (Figs. S1 and S2).

We found that the type of *PIK3CA* mutation had no differential effect on RFI (Fig. S3). Five-year RFI of patients with *PIK3CA* mutation in exon 9 or exon 20 were 94% and 93%, respectively, in the training cohort (*P *= 0.50), and 79% and 92% (*P *= 0.37), respectively, in the validation cohort. Notably, no recurrence occurred for the three patients in the CIT cohort whose tumor harbored concomitant *PIK3CA* exon 9 and exon 20 mutations.

We performed a multivariate analysis, adjusting for all significant prognostic variables (*P *≤ 0.05) including TNM stage and *PIK3CA* mutations (Table5). Risk of recurrence remained significantly lower in the training cohort for *PIK3CA*-mutated MSS CC patients as compared with wild-type *PIK3CA* MSS CC patients (HR = 0.12; 95% CI, 0.029–0.48; *P *= 0.0027). Although not significant, a trend toward decreased recurrence risk was observed for *PIK3CA-*mutated MSS CC patients in the validation cohort (HR = 0.49; 95% CI, 0.23–1.1; *P *= 0.074), and *PIK3CA* mutation variable remained with TNM stage in a backward–forward step selection to reduce the multivariate model to the only informative variables.

**Table 5:** Cox proportional hazards model for RFI among microsatellite stable tumors in the two cohorts

Variables	Univariate analysis					Multivariate analysis^1^			
	*n*	*n* events	HR	95% CI	*P* value	*n*	HR	95% CI	*P* value
*CIT cohort*									
TNM stage									
II	366	99	5.1	0.7–38	0.11	366	5.4	0.74–39	0.096
III	366	99	11	1.5–77	0.019		11	1.5–80	0.017
*PIK3CA*									
Mutated	366	99	0.12	0.029–0.48	0.0029		0.12	0.029–0.48	0.0027
Gender									
Male	366	99	1.1	0.73–1.6	0.67				
Age									
–	365	99	1	0.98–1	0.84				
Tumor location									
Proximal colon	366	99	1	0.66–1.5	0.99				
*KRAS*									
Mutated	363	99	1.2	0.81–1.8	0.37				
*BRAF*									
Mutated	323	78	1.5	0.36–6	0.6				
CIMP									
CIMP+	310	74	0.98	0.45–2.1	0.95				
*Dijon cohort*									
TNM stage									
II	327	97	2.5	0.89–6.8	0.084	327	2.5	0.89–6.8	0.084
III	327	97	4.2	1.5–12	0.0058		4.2	1.5–12	0.0058
*PIK3CA*									
Mutated	327	97	0.45	0.21–0.97	0.042		0.49	0.23–1.1	0.074
Gender									
Male	327	97	1.1	0.71–1.6	0.77				
Age									
-	327	97	0.99	0.98–1	0.46				
Tumor location									
Proximal colon	327	97	0.7	0.44–1.1	0.11				
*KRAS*									
Mutated	324	95	1.3	0.86–1.9	0.22				
*BRAF*									
Mutated	327	97	0.31	0.043–2.2	0.24				
CIMP									
CIMP+	326	97	0.89	0.45–1.8	0.75				

CIMP, CpG island methylator phenotype; HR, hazard ratio; CI, confidence interval; *P* value, Wald test *P* value.

1 Multivariate models include significant variables (*P* < 0.05).

Within the MSI tumors, patients with *PIK3CA* mutation in the training cohort experienced a significantly decreased RFI than those without *PIK3CA* mutation on univariate analysis (5-year RFI 67% vs. 87%, Log-rank *P *= 0.043, HR = 3.4; 95% CI, 0.96–12; *P* = 0.058) (Fig. S4A). This finding was not confirmed in the validation cohort (5-year RFI 100% vs. 80%, Log-rank *P *= 0.44) (Fig. S4B).

In the two cohorts, we found that the impact of *PIK3CA* mutation was not different between patients with MSS CC who received adjuvant chemotherapy and those who had no adjuvant treatment (Fig. S5).

## Discussion

Over the past several decades, significant progress has been achieved in the treatment of CC, mostly due to improvements in surgical techniques and chemotherapeutic regimens 31,32. These advances have contributed to increase cancer-specific survival (CSS), but patient outcome is still difficult to predict. Thus, prognostic biomarkers are required to guide physicians for patient management and follow-up after CC curative resection. Currently, the most recognized prognostic factor in CRC is the AJCC/UICC TNM staging system, defined by the depth of bowel wall invasion and by the presence of metastases in lymph nodes or more distant sites. However, there remains considerable heterogeneity in outcome within the different stages of this classification 33. Many studies assessed the potential prognostic impact of several somatic mutations including *KRAS*, *BRAF,* and *TP53* mutations after curative surgery 34–36. So far, none of these has been identified as a reproducible prognostic biomarker, except V600E *BRAF* mutation, which seems to be an independent biomarker of poor prognosis in MSS stage III CCs 37. Regarding *PI3KCA* mutations, reports are scarce and results are equivocal 16–20. None used an independent validation group to support their conclusions. Some of them included patients with stage IV disease 16,18,19, while others evaluated also patients with rectal cancer 16,18,19,21. In a series of 418 CRCs, Abubaker and colleagues reported that *PIK3CA* mutations were not associated with OS 16. Day and colleagues found similar results in a series of 585 stage II–III CRC regarding disease-free survival (DFS) 21. Nevertheless, this finding is in contradiction with other publications showing that any *PIK3CA* mutation induced a significant decrease in survival 17,18. Furthermore, studies that evaluated disease outcome according to the type of *PIK3CA* mutation have led to different conclusions. Fariña Sarasqueta and colleagues found that mutations located in exon 20 conferred poorer survival in stage III patients 20. But recently, Liao and colleagues emphasized that the prognostic impact of *PI3KCA* mutations was only restricted to the small proportion of CRCs harboring concomitant mutations in both exon 9 and 20 19. Finally, in a large series of 627 stage III CC, Ogino and colleagues found that *PIK3CA* mutation was neither a prognostic biomarker nor a predictive biomarker of response to adjuvant chemotherapy 22. It should be noted that these patients have been enrolled in a randomized controlled trial and received either the Roswell Park regimen of 5-fluoro-uracil (FU)/leucovorin (LV) or the regimen of irinotecan/FU/LV. These adjuvant treatments are, however, not those recommended in case of stage III disease (i.e., a combination of FU and oxaliplatin) 38.

We found in the present study that *PI3KCA* mutations had a favorable prognostic impact in MSS stage I–III CC. This finding is based on two large homogenous groups of patients, excluding those with rectal cancer or distant metastatic disease. Indeed, prognosis and management of patients with rectal cancer differ from that of patients with CC, as neoadjuvant chemoradiotherapy and quality of surgery have a significant impact on local recurrence 39,40. Moreover, prognostic biomarkers and chemotherapy regimens differ greatly between localized and advanced CC. Especially, targeted therapies can be used for patients with stage IV CRC, and discussions are still ongoing as to whether *PIK3CA* mutations including those present at the exon 20 are predictive biomarkers of response to anti-EGFR monoclonal antibodies 41.

Although retrospective, our training cohort showed quite similar molecular features than those of our prospective validation cohort, either for all patients or for patients with MSS CC. The mutation rates of *PIK3CA*, *KRAS,* and *BRAF* were consistent with those reported in the literature and were obtained from frozen tumoral tissues 2. In the literature, *PIK3CA* mutations have been reported to be significantly more frequent in women 24, in elderly patients 21, in proximal tumors 21,24, in *KRAS*-mutated tumors, 17,21,24 and in MSI tumors 16. In contrast with these reports, we did not find a consistent association between *PIK3CA* mutations and one particular clinical or molecular characteristic in our two cohorts of patients. The fact that the prognostic value of *PIK3CA* mutations was not confirmed on multivariate analysis could be explained by the clinical characteristics of the patients included in the validation cohort. The MSS tumors included in this second cohort were associated with a spontaneous better prognosis with significantly less stage III CC and less indications for adjuvant chemotherapy. One might assume that this group was underpowered to detect a statistical difference.

Very recently, Liao and colleagues highlighted that the use of aspirin after diagnosis among patients with *PIK3CA*-mutated CRC was associated with a significant longer CCS and OS compared with patients with *PIK3CA-*wild-type CRC, with an 82% reduction in CRC deaths and a 45% reduction in deaths from all causes 42. In this study based on two large prospective combined cohorts, the authors concluded that this gene could be used as a predictive biomarker for the prescription of aspirin therapy in adjuvant setting. Similarly, Domingo and colleagues also found in a large randomized trial comparing rofecoxib with placebo after primary CRC resection that regular use of low-dose aspirin after CRC diagnosis was associated with a reduced rate of recurrence in patients with *PIK3CA*-mutated tumors compared with *PIK3CA*-wild-type tumors (HR = 0.11; 95% CI; 0.001–0.832; *P* = 0.027) 43. But, in a series of 1487 CRC patients including 185 patients with *PIK3CA*-mutated tumors, Kothari and colleagues did not confirm the relationship between aspirin use and improved survival in patients with stage II-III disease 44. We were not able to assess patients’ survival according to *PIK3CA* status and aspirin treatment since information regarding aspirin therapy was not available in our database. However, definitive conclusion about the predictive value of *PIK3CA* mutations for aspirin treatment in nonmetastatic CRC can only be given with the results of a randomized trial.

The negative prognostic impact of *PIK3CA* mutations in MSI CC was not confirmed in the validation cohort. MSI CRCs have a significantly better prognosis with higher survival rates compared to MSS CRCs 23. In adjuvant setting, this phenotype is associated with a halving of the risk of recurrence 45. Therefore, for clinical practice, effective and accurate biomarkers of disease relapse after curative surgery are particularly required for patients with MSS tumors. MSI CRCs also constitute a heterogeneous group of CC, including both tumors with germline mutation of mismatch repair genes and tumors with CIMP and hypermethylation of the *MLH1* gene promoter. The two cohorts were too small to analyze the influence of *PIK3CA* mutations according to the different subgroups of MSI tumors.

As for the study of Ogino and colleagues, we found that *PIK3CA* mutations were not a predictive biomarker for response to adjuvant chemotherapy 22. In our study, *PIK3CA* mutation was a good prognostic biomarker, either in patients with MSS CC treated with adjuvant chemotherapy or those without adjuvant treatment. The type of adjuvant chemotherapy regimen was not recorded in our study, but it seems likely that a majority of patients received an oxaliplatin-based regimen, as recommended.

Our results seem to be in complete contrast with previous reports in CRC. Indeed, in experimental models, *PIK3CA* gain-of-function mutations have been shown to cause increased phosphorylation of AKT, aberrant activation of the PI3K/AKT/mTOR signaling pathway, and to promote oncogenic transformation. One would expect that proto-oncogene activation (or tumor suppressor gene inactivation) would clinically be associated with aggressive tumor behavior and unfavorable prognosis. However, in understanding of cancer biology, such reasoning seems too simplistic and contradicted by the well-known example of MSI phenotype in CRC 23. Finally, the good prognostic value of *PIK3CA* mutations has been emphasized in other cancer types, such as breast cancer, endometrial cancer, ovarian clear cell carcinoma, and esophageal squamous cell carcinoma 46–49. Notably, Kalinsky and colleagues showed in a series of 509 primary breast tumors with a median follow-up of more than 12 years that patients with *PIK3CA*-mutated tumors had a less aggressive phenotype with a significant improvement in OS and CSS 46. Similarly, Shigaki and colleagues found in a series of 219 patients who had undergone curative resection of stage I–III esophageal squamous cell carcinoma that patients with *PIK3CA* mutations experienced significantly longer DFS, CSS, and OS than those with wild-type *PIK3CA* 49. One possible explanation is that *PIK3CA* mutations could result in oncogene-induced senescence 46, but the biological mechanisms underlying this effect are still unclear. Finally, in CRC, Baba and colleagues reported in a series of 717 samples that phosphorylated AKT expression was significantly associated with *PIK3CA* mutations, and that patients with AKT-activated tumors had a significantly improved CSS in multivariate analysis 50. Surprisingly in this study, the authors used the data from the Nurses’ Health Study and the Health Professionals Follow-up Study, which are the same cohorts as those used in the studies of Ogino and colleagues and Liao and colleagues that led to diametrically opposite conclusions regarding the prognostic impact of *PIK3CA* mutations 17,19.

In summary, our study suggests that *PIK3CA* mutations are associated with better outcome in patients with resected MSS stage I-III CC. Our results may have clinical implications and provide useful information for the postoperative management of patients. Those with high-risk stage II or stage III *PIK3CA*-mutated MSS CC may not require adjuvant chemotherapy. Nevertheless, the mechanisms explaining this favorable prognostic impact of *PIK3CA* mutations remain to be elucidated. We cannot exclude that this could be the reflect of the predictive value of aspirin therapy. These results warrant confirmation in further translational studies.

## Supporting Information

**Figure S1.** Kaplan-Meier curves for relapse-free interval in the CIT cohort (A) and in the Dijon cohort (B), for overall survival in the CIT cohort (C) and in the Dijon cohort (D) according to *PIK3CA* status in stage I–II microsatellite stable tumors.

**Figure S2.** Kaplan-Meier curves for relapse-free interval in the CIT cohort (A) and in the Dijon cohort (B), for overall survival in the CIT cohort (C) and in the Dijon cohort (D) according to *PIK3CA* status in stage III microsatellite stable tumors.

**Figure S3.** Kaplan-Meier curves for relapse-free interval in the CIT cohort (A) and in the Dijon cohort (B), according to *PIK3CA* exon-specific mutation in microsatellite stable tumors.

**Figure S4.** Kaplan-Meier curves for relapse-free interval in the CIT cohort (A) and in the Dijon cohort (B), faccording to *PIK3CA* status in microsatellite instable tumors.

**Figure S5.** Kaplan-Meier curves for relapse-free interval according to *PIK3CA* status in microsatellite stable tumors for patients operated on without adjuvant chemothrapy in the CIT cohort (A) and in the Dijon cohort (D), and for those treated with adjuvant chemotherapy in the CIT cohort (B) and in the Dijon cohort (D).
