# Supplementary figures and images for: PIK3CA mutations predict recurrence in localized microsatellite stable colon cancer
Source: Cancer Med. 2015 Feb 2;4(3):371–82. doi: 10.1002/cam4.370 (PMC4380963; doi:10.1002/cam4.370)

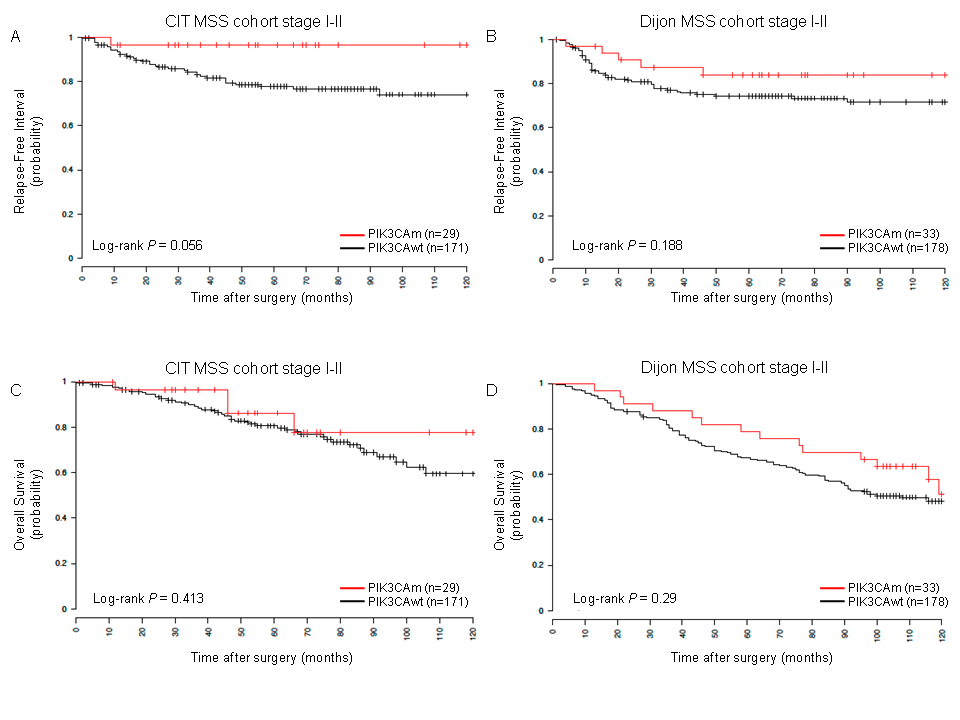

Supplement: Supplementary file 1 [file cam40004-0371-sd1.tif]

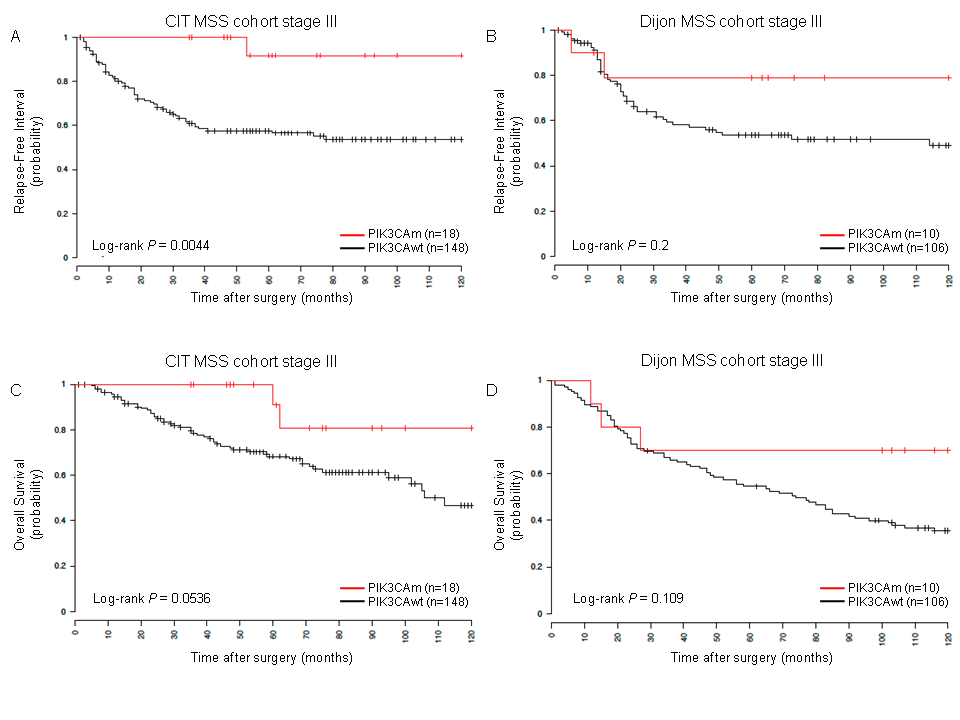

Supplement: Supplementary file 2 [file cam40004-0371-sd2.tif]

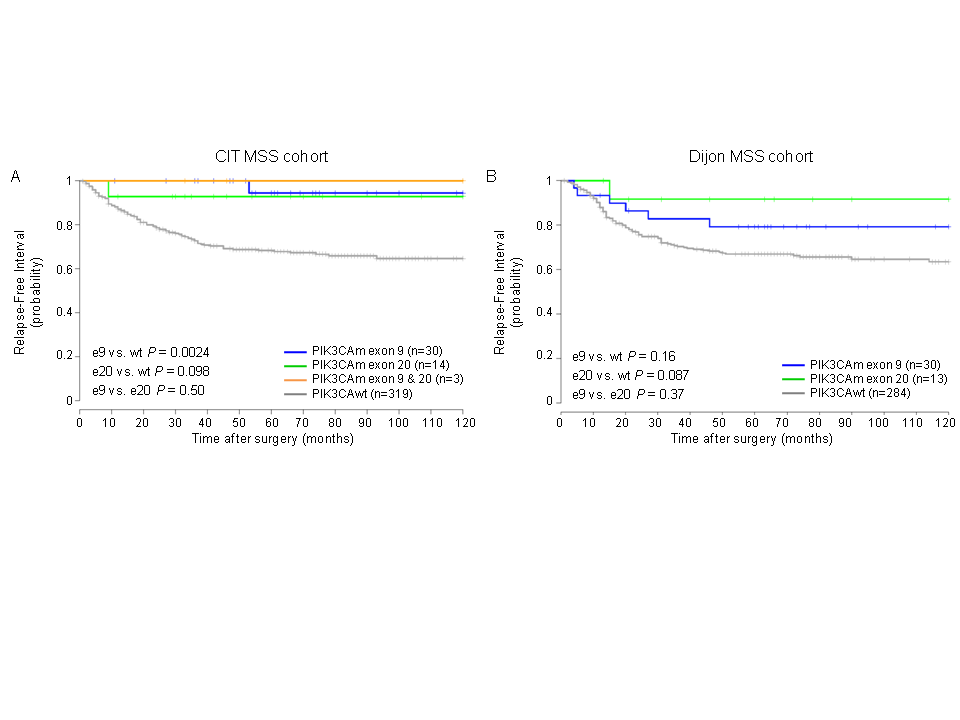

Supplement: Supplementary file 3 [file cam40004-0371-sd3.tif]

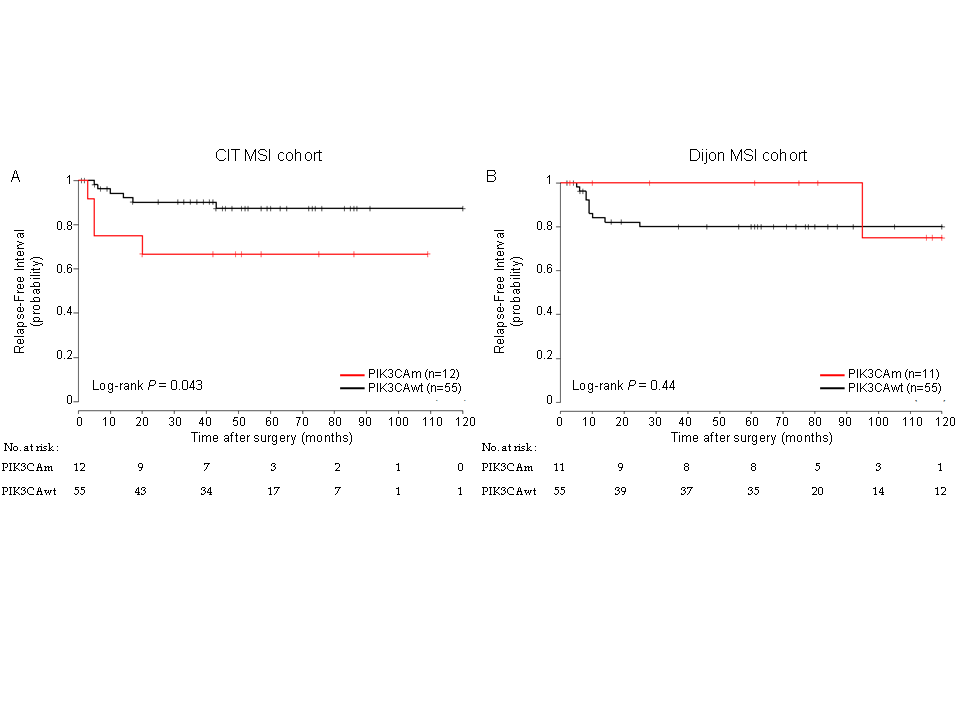

Supplement: Supplementary file 4 [file cam40004-0371-sd4.tif]

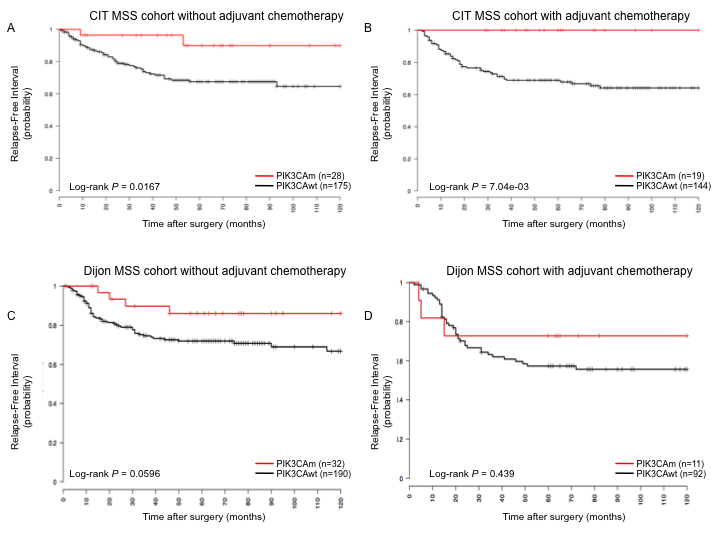

Supplement: Supplementary file 5 [file cam40004-0371-sd5.tif]
